# Global, regional, and national trends in the incidence of pneumoconiosis among populations aged 20 and above from 1990 to 2021

**DOI:** 10.3389/fpubh.2025.1608109

**Published:** 2025-08-18

**Authors:** Qiyun Cheng, Shiyue He, Yongbin Hu, Pinhua Pan, Xinyue Hu, Shuya Liao, Shunjun Wang, Hui Li

**Affiliations:** ^1^Department of Pathology, School of Basic Medical Science, Central South University, Changsha, Hunan, China; ^2^Department of Pathology, Xiangya Hospital, Central South University, Changsha, Hunan, China; ^3^Center of Respiratory Medicine, Xiangya Hospital, Central South University, Changsha, Hunan, China; ^4^Hunan Engineering Research Center for Intelligent Diagnosis and Treatment of Respiratory Disease, Changsha, China; ^5^National Key Clinical Specialty, Branch of National Clinical Research Center for Respiratory Disease, Xiangya Hospital, Central South University, Changsha, China; ^6^National Clinical Research Center for Geriatric Disorders, Xiangya Hospital, Changsha, Hunan, China; ^7^Yali High School International Department, Changsha, Hunan, China; ^8^Department of Cardiology Surgery, Xiangya Hospital, Central South University, Changsha, China; ^9^Department of Respiratory and Critical Care Medicine, The First Hospital of Changsha, The Affiliated Changsha Hospital of Xiangya School of Medicine, Central South University, Changsha, Hunan, China

**Keywords:** pneumoconiosis, incidence, global burden of diseases, age-standardized incidence rate, estimated annual percentage change

## Abstract

**Background:**

Pneumoconiosis remains one of the most critical occupational health hazards globally. Utilizing data from the Global Burden of Disease (GBD) 2021, we have updated the epidemiological trends of pneumoconiosis.

**Methods:**

We conducted and analyzed pneumoconiosis-related data from the GBD 2021 study for individuals aged ≥20 years. Our analysis described the incident cases and age-standardized incidence rates (ASIRs) across various global regions and age groups. Temporal trends were evaluated using Estimated Annual Percentage Change (EAPC) for ASIRs between 1990 and 2021.

**Results:**

The ASIR of pneumoconiosis among individuals aged ≥20 years declined globally at an annual average of 0.48% between 1990 and 2021. Except for high socio-demographic index (SDI) regions, the ASIR of pneumoconiosis declined across all other SDI categories. Males had significantly higher incidence rates than females, especially in older adults. Silicosis emerged as the predominant type of pneumoconiosis, constituting ~56.7% of cases. While the ASIRs for silicosis, coal workers' pneumoconiosis, and other pneumoconiosis decreased, the ASIRs for asbestosis exhibited a notable upward trend, with an EAPC of 1.21%. A strong negative correlation was observed between the EAPC of pneumoconiosis incidence and the 1990 ASIRs values. Notably, the EAPC showed a statistically significant but very weak positive correlation with the 2021 Human Development Index (HDI) values.

**Conclusion:**

Despite a gradual global decline in the ASIR of pneumoconiosis, the disease burden remains substantial in certain regions. Our findings could inform governments and policymakers in developing targeted prevention strategies to mitigate this burden. Future strategies should integrate technological innovation with regulatory frameworks, prioritizing male-dominated high-risk sectors through strengthened global asbestos bans and lifetime health surveillance for workers in pneumoconiosis-prone occupations worldwide.

## 1 Introduction

Pneumoconiosis refers to a group of lung diseases caused by the inhalation and subsequent deposition of organic or more commonly, inorganic dust particles in the lungs (1). The most prevalent types of dust exposure linked to this condition include asbestosis, silicosis and coal workers' pneumoconiosis (commonly known as coal miner's lung) (2). These particulate materials provoke chronic pulmonary inflammation and fibrosis, leading to the development of irreversible lung damage characteristic of pneumoconiosis (3). Typically, individuals are exposed to these particulates and fibers in the workplace environment, which is why it is classified as an occupational disease. This debilitating condition can cause severe respiratory dysfunction, diminished quality of life, elevated susceptibility to immune-related diseases such as tuberculosis and lung cancer. In severe cases, it may ultimately result in death from respiratory or heart failure (4, 5).

According to the latest joint estimates by the world health organization (WHO) and the International Labor Organization (ILO) on the work-related burden of disease and injury, occupational exposure to hazardous substances accounts for staggering global health impacts. The data reveal asbestos exposure alone caused 20,981 deaths, while silica dust exposure resulted in 42,258 fatalities (6). These occupational hazards correspond to 3.97 and 1.3 million disability-adjusted life years (DALYs) (6). Pneumoconiosis poses a multifaceted societal burden, compromising respiratory health while simultaneously straining healthcare infrastructure, hindering workforce productivity, and generating substantial economic losses. Empirical evidence from China illustrates this impact: annual direct medical expenditures associated with the disease reached 8 billion Chinese yuan (CNY), with indirect productivity losses estimated at 20 billion CNY in 2013 (7). Therefore, these imperatives underscore that prevention and clinical treatment of pneumoconiosis should be prioritized within national economic agendas and population health frameworks.

Nevertheless, contemporary therapeutic approaches for pneumoconiosis remain constrained by critical limitations: invasive procedures such as lung transplantation are severely restricted by donor scarcity (affecting <5% of eligible patients), while palliative interventions including therapeutic lung lavage show negligible survival benefits, as evidenced by a multicenter cohort study (8). This compelling evidence highlights the imperative for prioritizing primary prevention strategies within global health agendas. An analysis of the epidemiological distribution and its underlying determinants across countries and regions is fundamental to identify critical gaps in existing prevention strategies and promote more effective and evidence-based interventions. Specifically, a comprehensive assessment of the global and national burden of pneumoconiosis is crucial for evidence-based decision-making and optimizing resource allocation to the most urgent public health priorities. In this study, utilizing data reported in the Global Burden of Disease (GBD) 2021 study, we present the global incidence of pneumoconiosis and four sub-type pneumoconiosis and analyze their temporal trends from 1990 to 2021 among individuals over 20 years old at the global, regional and national levels.

## 2 Methods

### 2.1 Study data

The GBD 2021 study provides a comprehensive epidemiologic evaluation of 369 diseases and injuries, 286 causes of death, and 87 risk factors across regions, countries, sexes, and etiologies. The GBD 2021 study encompasses data from 204 countries, seven super-regions and 21 geographical regions, spanning the period from 1990 to 2021. The methodology employed in the GBD 2021 study, led by the Institute for Health Metrics and Evaluation (IHME), has been extensively documented in prior publications (9). Detailed data on both fatal and non-fatal outcomes were retrieved using the Global Health Data Exchange (GHDx) query tool, accessible at http://ghdx.healthdata.org/gbd-results-tool. For this study, the extracted data included incident number, age-standardized incidence rate (ASIR), and the percentage change in incident number and ASIR from 1990 to 2021, analyzed at global, regional, national, and cause-specific levels. The study specifically examined the etiologies of pneumoconiosis, including silicosis, asbestosis, coal workers' pneumoconiosis (CWP), and other forms of pneumoconiosis (10). To estimate the proportion of pneumoconiosis attributable to these four etiologies, the study utilized DisMod-MR 2.1, a Bayesian meta-regression tool. We first ran a single pneumoconiosis model, grouping together all the pneumoconiosis data (asbestosis, coal worker's pneumoconiosis, silicosis, and other pneumoconiosis) and ran a single DisMod model. We set remission to 0 and assumed no prevalence or incidence before the age of 15. We include a predictive covariate on healthcare access and quality. Location random effects are set at −1 to 1 for prevalence. This single pneumoconiosis model estimated all-pneumoconiosis prevalence by year, age, sex and location. Based on the Socio-demographic Index (SDI), 204 countries and regions were stratified into five quintiles by sorting all entities in ascending order of SDI values and dividing them into equal-sized groups: low (0–0.45), low-middle (0.45–0.61), middle (0.61–0.69), high-middle (0.69–0.80) and high (0.80–1.00) SDI regions (11). Furthermore, the 2021 Human Development Index (HDI) for 187 countries and territories was obtained from the United Nations Development Programme. The HDI serves as a composite measure of a nation's health, education, and income levels, reflecting three key dimensions: a long and healthy life, access to knowledge, and a decent standard of living (9). In this study, we utilized the SDI to examine the association between pneumoconiosis prevalence and socioeconomic developmental gradients across diverse countries and regions. Concurrently, we employed the HDI to assess the comparative efficacy of various public health strategies implemented for pneumoconiosis prevention and treatment.

### 2.2 Statistical analysis

The study employed the ASIR and its estimated annual percentage change (EAPC) to assess trends in pneumoconiosis incidence from 1990 to 2021. Standardization of incidence rates was necessary to account for variations in age structures across different populations, enabling meaningful comparisons. ASIR trends also serve as a valuable indicator of shifting disease patterns within populations and offer insights into evolving risk factors. The EAPC, a widely used summary statistic, was utilized to evaluate changes in ASIR over a specified time interval. Detailed methodologies for calculating ASIR, EAPC and 95% uncertainty interval (UI) have been described in previous studies (12–14). To identify factors influencing EAPC, the study analyzed correlations between EAPC and ASIR in 1990, as well as between EAPC and HDI in 2021, at the national level. Additionally, a hierarchical cluster analysis was performed to categorize the 204 countries and regions into four groups based on their EAPCs and 95% Confidence intervals (CIs). All statistical analyses and visualizations were conducted using R software version 3.6.3, with a *P*-value of < 0.05 deemed statistically significant.

## 3 Results

### 3.1 Overview of the global burden of pneumoconiosis in individuals aged ≥20 years

**Figure 1:**
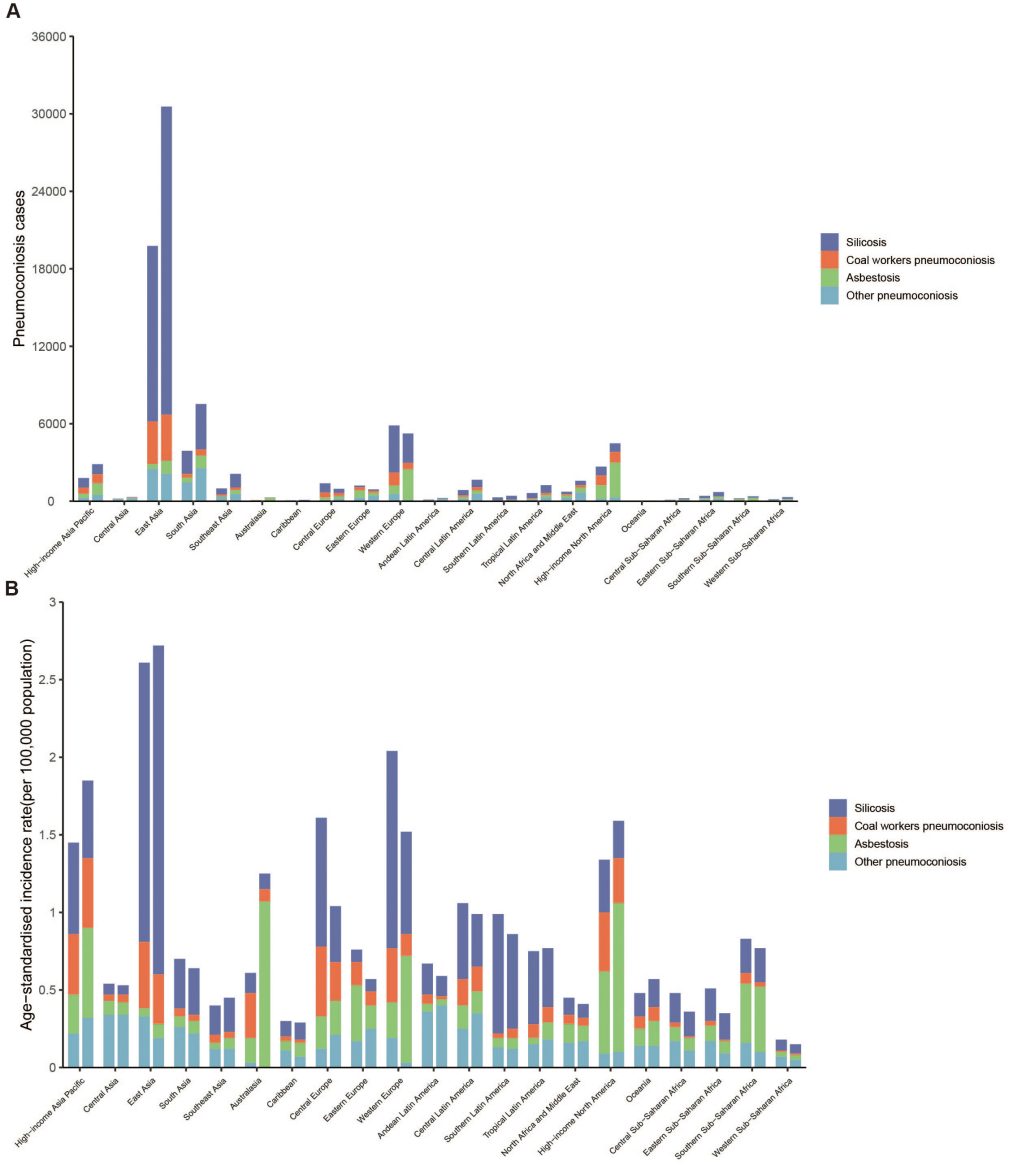
**(A)** Number of incident cases of pneumoconiosis at the 21 GBD regional levels in 1990 and 2021. **(B)** Age-Standardized Incidence Rate of pneumoconiosis at the 21 GBD regional levels in 1990 and 2021. For each group, the left column presents case data for 1990, while the right column presents case data for 2021.

**Figure 2:**
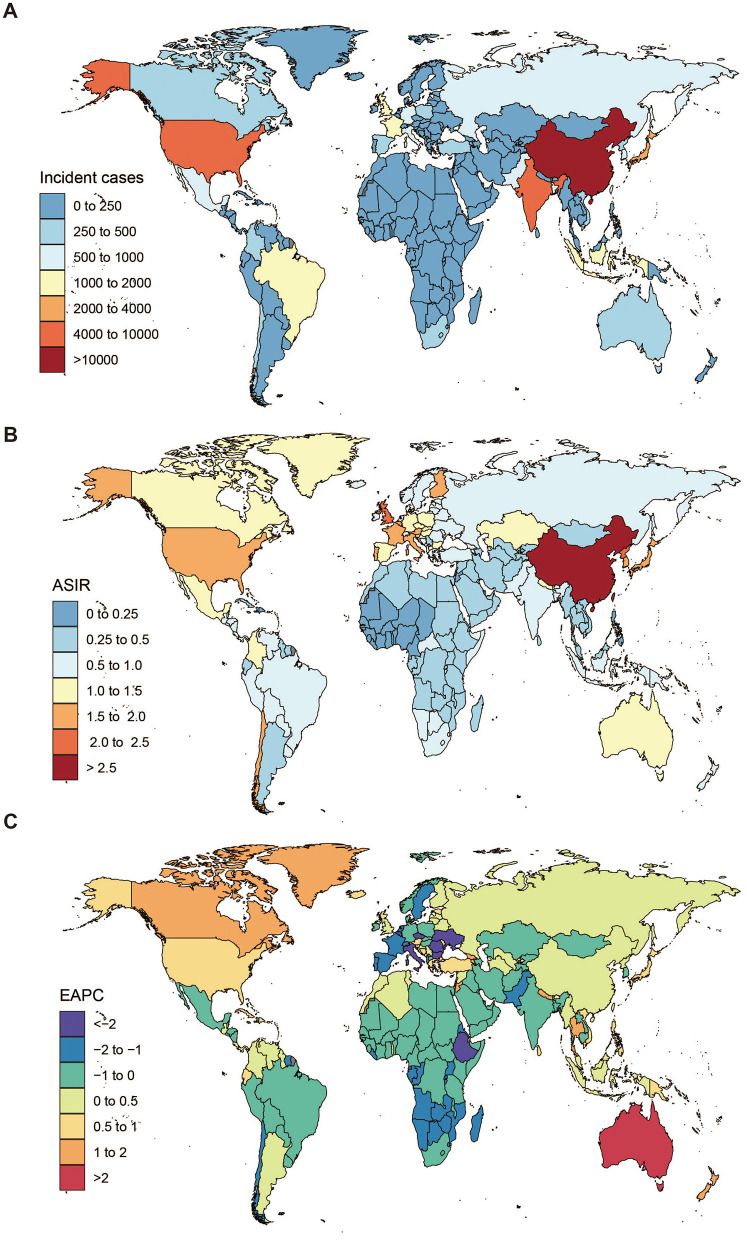
The global disease burden of pneumoconiosis across countries. **(A)** Incident cases of pneumoconiosis in 2021, **(B)** ASIR per 100,000 of pneumoconiosis in 2021, **(C)** EAPC in ASIR of pneumoconiosis from 1990 to 2021. ASIR, Age-Standardized Incidence Rate; EAPC, Estimated Annual Percentage Change.

The global incident cases of pneumoconiosis among individuals aged ≥20 years showed an increasing trend from 1990 to 2021 (Table 1). However, the global ASIR of pneumoconiosis in individuals aged ≥20 years declined from 1.35 (95% UI: 1.14–1.56) in 1990 to 1.18 (95% UI: 1.02–1.34) in 2021, with EAPC of −0.48% (95% UI: −0.57% to −0.40%; Table 1). High SDI region exhibited the highest ASIR of pneumoconiosis among individuals aged ≥20 years and was the only SDI stratum demonstrating an increasing ASIR trends from 1990 to 2021. In contrast, middle SDI region accounted for the highest number of incident cases (Supplementary Figure 1, Table 1). Regionally, East Asia, High-income Asia Pacific and High-income North America reported the highest ASIR values in 2021 (Figure 1, Table 1). From 1990 to 2021, six regions exhibited rising ASIR trends, with the most pronounced increases observed in Australasia (EAPC = 2.72), High-income North America (EAPC = 0.85) and Oceania (EAPC = 0.72; Table 1). Conversely, the sharpest declines in ASIR were noted in Eastern Sub-Saharan Africa, Eastern Europe and Central Europe (Figure 1, Table 1). Among the 204 countries assessed, Monaco and China recorded the highest ASIR in 2021, while Philippines, Islamic Republic of Mauritania and Nigeria reported the lowest rates (Figure 2, Supplementary Table 1). Over the study period, ASIR increased in 91 countries, with the most rapid rise in Australia (EAPC = 2.90), while Ukraine and Belgium had the most significant declines (Figure 2, Supplementary Table 1).

**Table 1:** The incident cases and ASIR of pneumoconiosis in individuals age ≥20 in 1990 and 2021.

**Characteristics**	**1990**		**2021**		**1990–2021**
	**Incident cases (95% UI)**	**ASIR per 100 000 (95% UI)**	**Incident cases (95% UI)**	**ASIR per 100 000 (95% UI)**	**EAPC (95% CI)**
Global	41,479 (35,116–47,972)	1.35 (1.14–1.56)	62,184 (53,682–70,514)	1.18 (1.02–1.34)	−0.48 (−0.57 to −0.40)
**Sex**					
Male	36,249 (30,871–41,987)	2.37 (2.02–2.75)	53,063 (46,071–60,255)	2.04 (1.77–2.32)	−0.54 (−0.64 to −0.43)
Female	5,230 (4,156–6,484)	0.34 (0.27–0.42)	9,121 (7,493–10,970)	0.34 (0.28–0.41)	−0.03 (−0.09 to −0.03)
**SDI**					
High SDI	10,248 (8,739–11,835)	1.63 (1.39–1.88)	14,223 (12,576–16,019)	1.65 (1.46–1.86)	0.01 (−0.06–0.08)
High-middle SDI	12,062 (10,188–14,008)	1.74 (1.47–2.02)	16,004 (13,763–18,232)	1.60 (1.38–1.82)	−0.43 (−0.51 to −0.35)
Middle SDI	14,137 (11,780–16,666)	1.48 (1.23–1.74)	22,642 (19,273–25,895)	1.33 (1.13–1.52)	−0.38 (−0.52 to −0.23)
Low-middle SDI	3,840 (3,210–4,516)	0.67 (0.56–0.79)	7,072 (6,047–8,212)	0.61 (0.52–0.71)	−0.31 (−0.37 to −0.24)
Low SDI	1,160 (956–1,361)	0.52 (0.43–0.61)	2,215 (1,886–2,576)	0.42 (0.35–0.48)	−0.76 (−0.81 to −0.71)
**Region**					
Andean Latin America	127 (110–145)	0.67 (0.58–0.76)	250 (221–282)	0.59 (0.52–0.66)	−0.15 (−0.27 to −0.04)
Australasia	85 (75–95)	0.60 (0.54–0.68)	294 (271–325)	1.26 (1.16–1.39)	2.72 (2.51–2.93)
Caribbean	60 (47–75)	0.30 (0.23–0.37)	92 (71–117)	0.29 (0.22–0.36)	−0.11 (−0.20 to −0.03)
Central Asia	204 (168–246)	0.54 (0.45–0.65)	319 (270–378)	0.52 (0.44–0.62)	−0.23 (−0.29 to −0.17)
Central Europe	1,383 (1,193–1,591)	1.61 (1.39–1.85)	960 (828–1,115)	1.05 (0.90–1.22)	−1.30 (−1.38 to −1.23)
Central Latin America	861 (710–1,029)	1.05 (0.87–1.26)	1,656 (1,375–1,968)	0.99 (0.82–1.17)	−0.43 (−0.50 to −0.36)
Central Sub-Saharan Africa	112 (93–131)	0.47 (0.39–0.54)	228 (193–266)	0.36 (0.30–0.42)	−0.95 (−0.98 to −0.91)
East Asia	19,776 (16,466–23,396)	2.61 (2.17–3.09)	30,564 (26,169–35,201)	2.71 (2.32–3.12)	0.01 (−0.16–0.18)
Eastern Europe	1,207 (965–1,452)	0.76 (0.61–0.91)	915 (748–1,084)	0.57 (0.47–0.67)	−1.33 (−1.60 to −1.06)
Eastern Sub-Saharan Africa	406 (335–475)	0.51 (0.42–0.59)	708 (602–815)	0.36 (0.30–0.41)	−1.34 (−1.42 to −1.26)
High-income Asia Pacific	1,792 (1,520–2,073)	1.46 (1.24–1.68)	2,862 (2,530–3,245)	1.85 (1.64–2.10)	0.53 (0.42–0.64)
High-income North America	2,673 (2,169–3,188)	1.34 (1.09–1.60)	4,479 (3,843–5,164)	1.60 (1.37–1.84)	0.85 (0.54–1.17)
North Africa and Middle East	728 (577–927)	0.45 (0.36–0.57)	1,578 (1,256–1,951)	0.41 (0.32–0.50)	−0.07 (−0.32–0.17)
Oceania	15 (12–19)	0.48 (0.37–0.60)	43 (35–51)	0.57 (0.47–0.67)	0.72 (0.58–0.85)
South Asia	3,897 (3,247–4,585)	0.71 (0.59–0.83)	7,526 (6,406–8,820)	0.65 (0.55–0.76)	−0.26 (−0.36 to −0.17)
Southeast Asia	991 (764–1,274)	0.40 (0.31–0.52)	2,118 (1,660–2,648)	0.45 (0.35–0.56)	0.53 (0.40–0.67)
Southern Latin America	302 (269–337)	1.00 (0.89–1.12)	416 (375–464)	0.86 (0.78–0.96)	−0.61 (−0.78 to −0.45)
Southern Sub-Saharan Africa	219 (183–258)	0.84 (0.70–0.99)	376 (324–429)	0.77 (0.66–0.88)	−0.75 (−1.02 to −0.47)
Tropical Latin America	630 (524–752)	0.76 (0.63–0.90)	1,243 (1,079–1,409)	0.77 (0.67–0.88)	−0.29 (−0.50 to −0.09)
Western Europe	5,864 (5,102–6,651)	2.05 (1.78–2.33)	5,242 (4,742–5,816)	1.52 (1.37–1.68)	−1.11 (−1.16 to −1.06)
Western Sub-Saharan Africa	148 (111–195)	0.17 (0.13–0.23)	313 (232–416)	0.14 (0.10–0.19)	−0.65 (−0.71 to −0.60)

**Figure 3:**
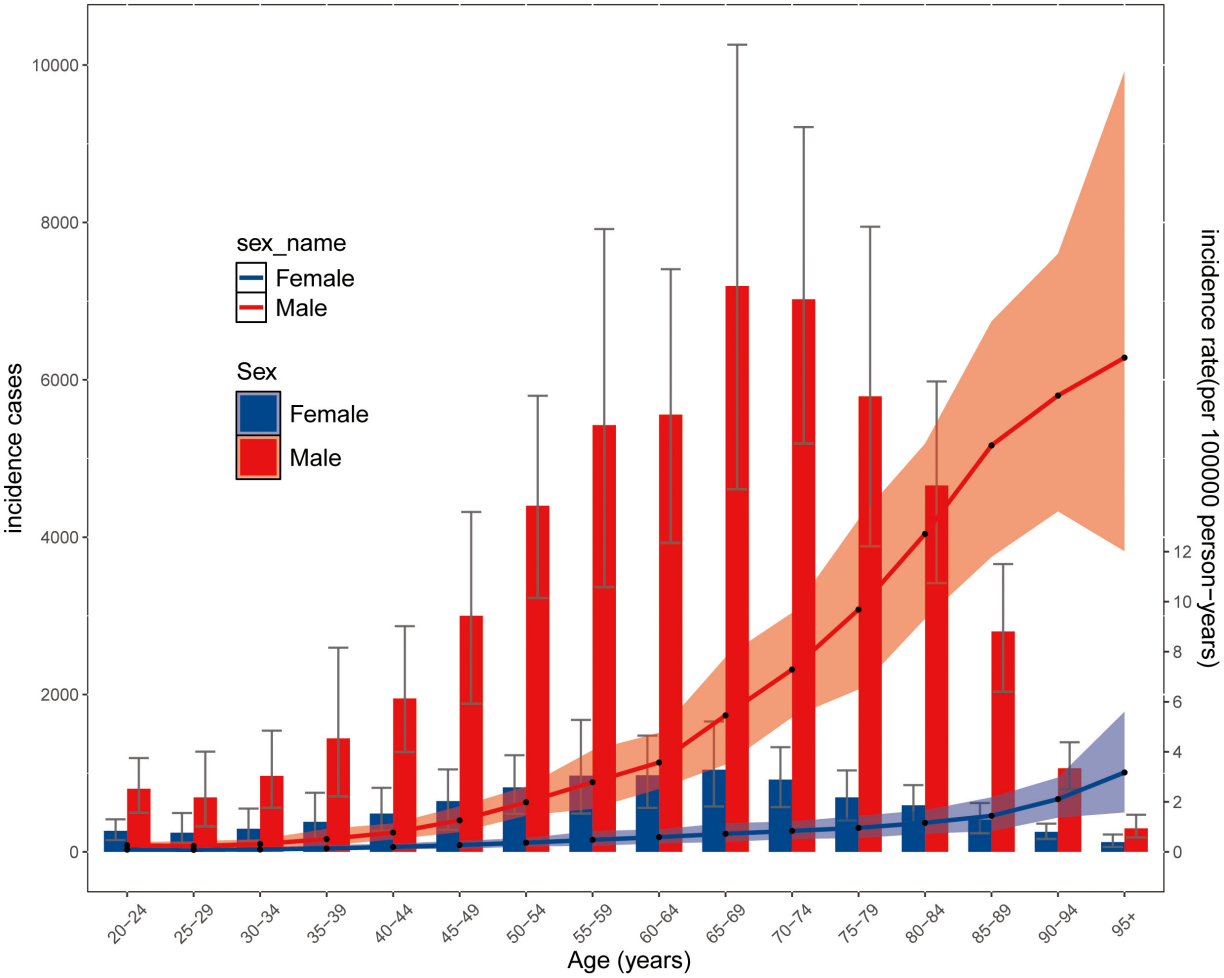
Global cases and age-standardized rates of incidence of pneumonocosis per 100,000 population by age and sex, 2021. Detailed data are provided in Supplementary Table 2. Shading indicates the upper and lower limits of the 95% uncertainty intervals (95% UIs).

Age-specific incidence cases exhibited a bell-shaped distribution peaking at 65–69 years, with a similar peak age observed in both sexes. Incidence rates increased with age in both sexes and, with sharper rise in male populations post 60–64 years (Figure 3, Supplementary Table 2). Males consistently had higher absolute incidence counts than females across all age groups (Figure 3, Supplementary Table 2).

**Figure 4:**
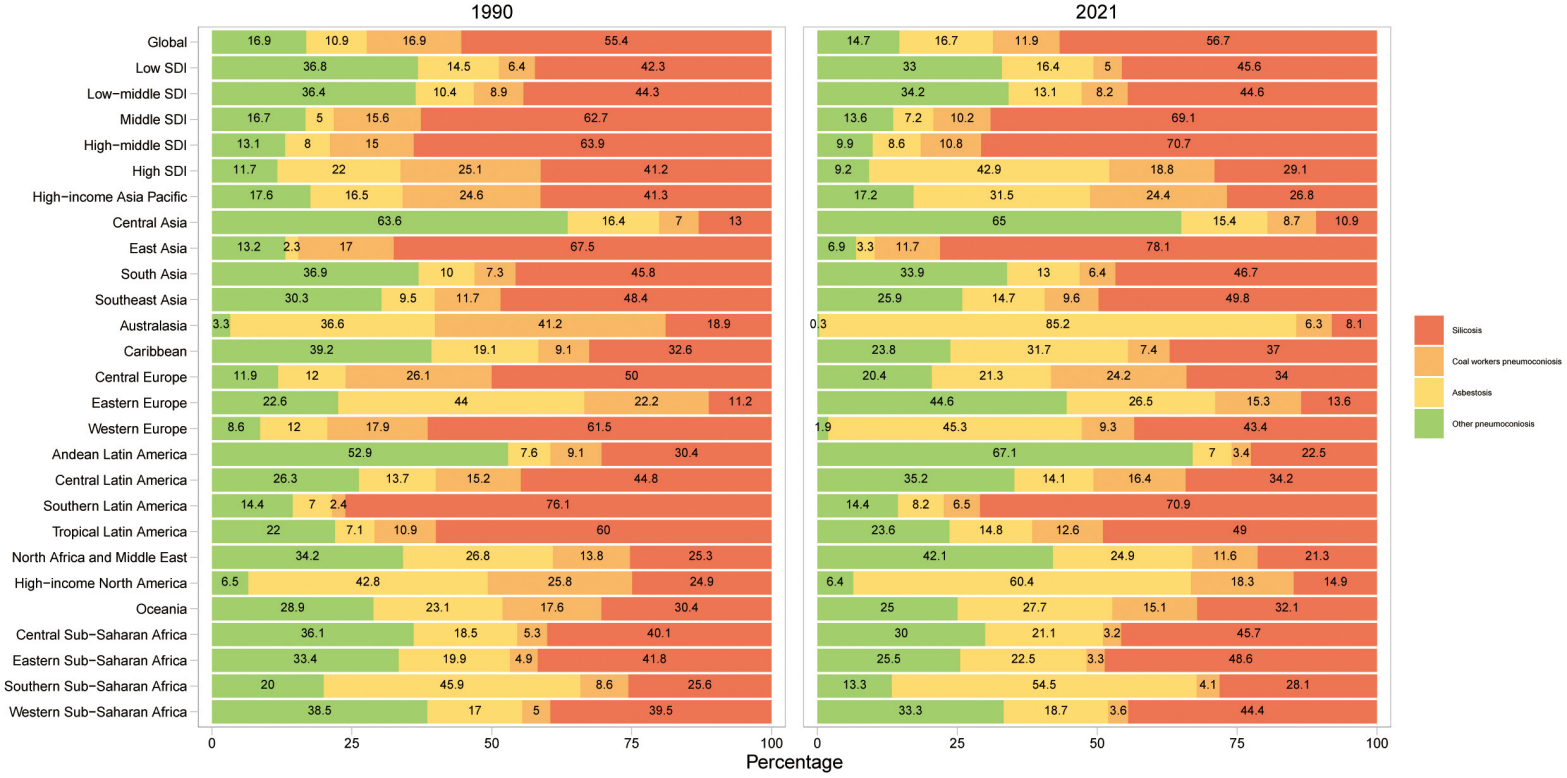
Contribution of silicosis, coal workers pneumoconiosis, asbestosis, and other pneumoconiosis to pneumonocosis incident cases, by regions for both sexes combined in 1990 and 2021. SDI, sociodemographic index.

**Figure 5:**
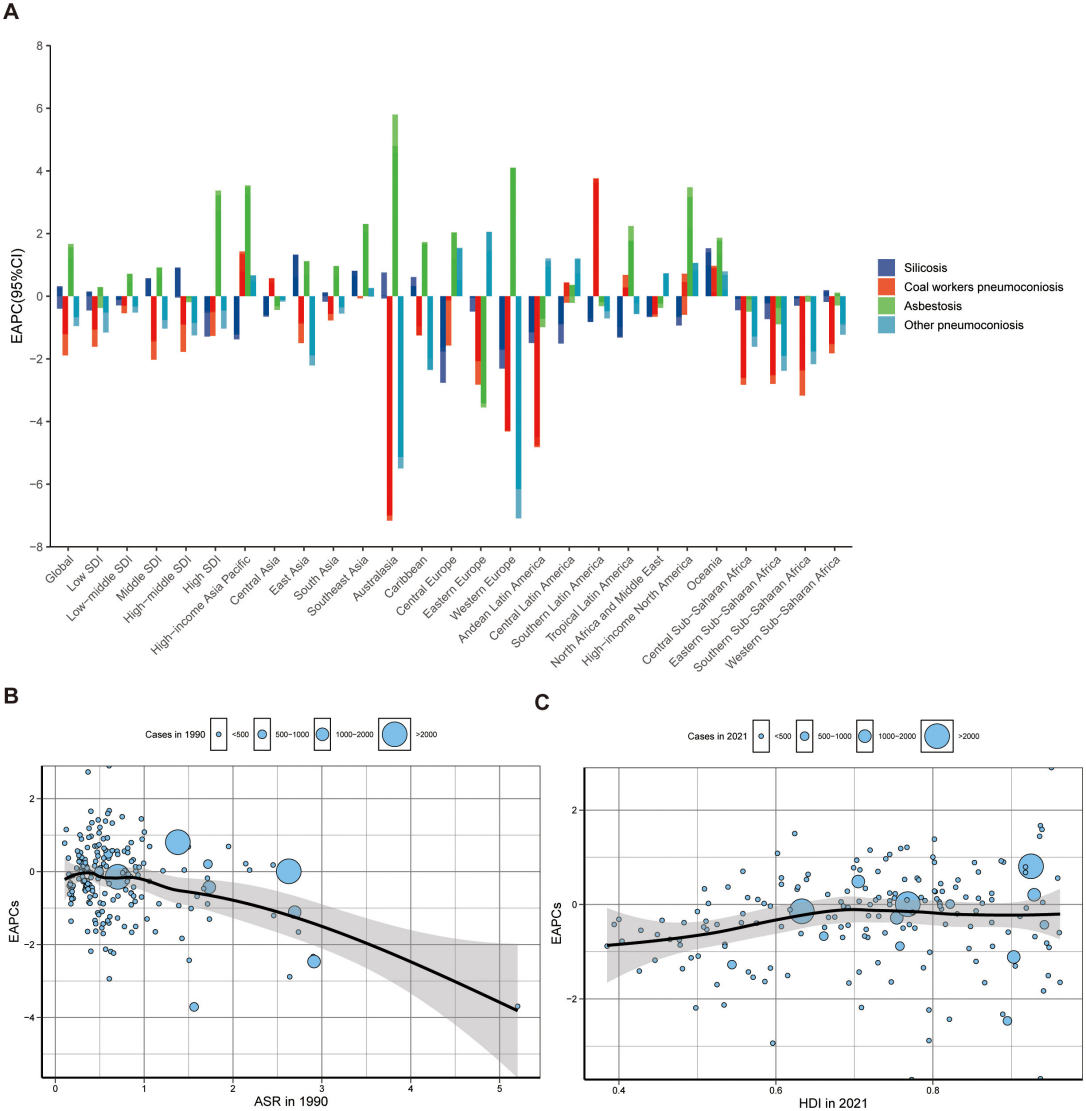
The EAPCs of pneumoconiosis at global and regional level. **(A)** The EAPC of pneumoconiosis ASIR from 1990 to 2021, both region and by etiologies. The correlation between EAPC and **(B)** ASIR in 1990 (*t* = −5.2594, df = 202, *P* = 3.67e-07, 95% CI: −0.46 to −0.22, *r* = −0.35) and **(C)** HDI in 2021 (*t* = 2.17, df = 178, *P* = 0.03, 95% CI: 0.01–0.30, *r* = 0.16). The circles represent countries that were available on HDI data. The size of the circle is increased with the cases of pneumoconiosis. The ρ indices and *P* values presented in **(B)** and **(C)** were derived from Pearson correlation analysis. EAPC, Estimated annual percentage change; ASIR, Age-standardized incidence rate; HDI, Human development index; ASR, Age-standardized rate.

Silicosis and asbestosis remained the leading causes of pneumoconiosis, accounting for over 50% of cases (Figure 4; Supplementary Table 3). Notable shifts included a decline in silicosis in high SDI regions (41.2%−29.1%) and Western Europe (61.5%–43.4%), alongside a surge in asbestosis in Australasia (36.6%−85.2%) and Western Europe (12%−45.3%; Figures 4 and 5A). Figures 5B, C illustrate significant associations between EAPC, ASIR (1990), and HDI (2021; Supplementary Table 4). EAPC demonstrated a negative correlation with ASIR in 1990 (*r* = −0.35, 95% CI: −0.46 to −0.22), indicating the impact of baseline disease burden. Conversely, statistically significant but very weak positive correlation was observed with 2021 HDI, reflecting healthcare accessibility (*r* = 0.16, 95% CI: 0.01–0.30) (12).

Subtype-specific patterns varied: silicosis showed negative correlations with both ASIR (1990) and HDI (2021; Supplementary Figure 2). Asbestosis showed no correlation with ASIR (1990), but a positive association with HDI (2021; Supplementary Figure 3). CWP showed negative but very weak correlation with ASIR (1990), but no correlation with HDI (2021; Supplementary Figure 4). Other pneumoconiosis showed negative correlation with ASIR (1990), but no significant association with HDI (2021; Supplementary Figure 5).

### 3.2 Pneumoconiosis among individuals aged ≥20 caused by silicosis

In 2021, silicosis accounted for 56.7% of pneumoconiosis among individuals aged ≥20 (Figure 4, Supplementary Table 5), with proportions exceeding 60% in East Asia and Southern Latin America. China reported the highest silicosis incidence cases, while Tokelau recorded the lowest (Supplementary Figure 6A, Supplementary Table 5). The highest ASIR were observed in China, followed by Monaco and Chile, while the lowest ASIR were in Palestine and State of Kuwait (Supplementary Figure 6B, Supplementary Table 5).

From 1990 to 2021, global silicosis ASIR demonstrated a decreasing trend (EAPC = −0.40), with declines in all SDI regions except middle-SDI (Supplementary Table 5). Regionally, Central Europe experienced the most substantial ASIR decline; while Oceania recorded the highest ASIR increase (Figure 5A, Supplementary Table 5). At the country level, the most pronounced reductions were observed in Norway, Kuwait and Bulgaria (Supplementary Figure 6C, Supplementary Table 5).

### 3.3 Pneumoconiosis among individuals aged ≥20 caused by coal workers pneumoconiosis

In 2021, CWP accounted for 11.9% of pneumoconiosis cases among individuals aged ≥20 (Figure 4, Supplementary Table 6). Six regions, including High-income Asia Pacific, Central Europe, Eastern Europe, Central Latin America, High-income North America and Oceania, had >15% CWP attribution (Figure 4, Supplementary Table 6). China recorded the highest incidence cases, whereas Tokelau had the lowest (Supplementary Figure 7A, Supplementary Table 6). The highest ASIR was in the Republic of Korea, Czech Republic and Belgium; while Belarus reported the lowest (Supplementary Figure 7B, Supplementary Table 6).

From 1990 to 2021, global CWP ASIR demonstrated a decreasing trend (EAPC = −1.89), with declines in all SDI regions (Supplementary Table 6). Regionally, Australasia experienced the most substantial ASIR decline (EAPC = −7.16), while Southern Latin America (EAPC = 3.61) recorded the highest ASIR increase (Figure 5A, Supplementary Table 6). At the country level, the most pronounced reductions were observed in Mauritius, Italy and Australia (Supplementary Figure 7C, Supplementary Table 6).

### 3.4 Pneumoconiosis among individuals aged ≥20 caused by asbestosis

In 2021, asbestosis accounted for 16.7% of pneumoconiosis cases among individuals aged ≥20 (Figure 4, Supplementary Table 7). Four regions, including Australasia, Western Europe, High-income North America and Southern Sub-Saharan Africa, had >40% asbestosis attribution (Figure 4, Supplementary Table 7). The United States of America recorded the highest incidence cases, whereas Tokelau had the lowest (Supplementary Figure 8A, Supplementary Table 7). The highest ASIR was in Finland, followed by United Kingdom and Australia, while the Maldives reported the lowest (Supplementary Figure 8B, Supplementary Table 7).

From 1990 to 2021, global ASIR of asbestosis exhibited a concerning upward trend, with an EAPC of 1.21 (Supplementary Table 7). All SDI regions demonstrated rising ASIR trends except high-middle SDI and low SDI regions (Supplementary Table 7). Regionally, Eastern Europe experienced the most substantial ASIR decline, while Australasia recorded the highest ASIR increase (Figure 5A, Supplementary Table 7). At the country level, the most pronounced reductions were observed in Lithuania, Qatar and Latvia (Supplementary Figure 8C, Supplementary Table 7).

### 3.5 Pneumoconiosis among individuals aged ≥20 caused by other pneumoconiosis

In 2021, other pneumoconiosis accounted for 14.7% of cases among individuals aged ≥20 (Figure 4, Supplementary Table 8). Seven regions, including Central Asia, South Asia, Eastern Europe, Andean Latin America, Central Latin America, North Africa and Middle East and Western Sub-Saharan Africa, had >30% attribution (Figure 4, Supplementary Table 8). India recorded the highest incidence cases, whereas Tokelau had the lowest (Supplementary Figure 9A, Supplementary Table 8). Taiwan (Province of China) had the highest ASIR, while Australia reported the lowest (Supplementary Figure 9B, Supplementary Table 8).

From 1990 to 2021, global ASIR of other pneumoconiosis demonstrated a decreasing trend (EAPC = −0.95), with declining ASIR trends across all SDI regions (Supplementary Table 8). Regionally, Western Europe experienced the most substantial ASIR decline (EAPC = −7.09), while Eastern Europe (EAPC = 1.44) recorded the highest ASIR increase (Figure 5A, Supplementary Table 8). At the country level, the most pronounced reductions were observed in the United Kingdom (Supplementary Figure 9C, Supplementary Table 8).

## 4 Discussion

Using GBD 2021 data, this study assessed the global, regional, and national burden of pneumoconiosis among individuals aged ≥20 from 1990 to 2021, analyzing silicosis, asbestosis, CWP and other pneumoconiosis. Pneumoconiosis, a severe occupational lung disease caused by chronic dust inhalation, remains a major global health concern, particularly in developing regions reliant on coal as a primary energy source (4, 15). Despite its global impact, epidemiological data on pneumoconiosis remain limited. While existing studies leveraging GBD 2021 data have mapped the global/regional pneumoconiosis burden from 1990 to 2021, our analysis extends beyond foundational burden assessments to specifically characterize disease patterns across four etiological forms of pneumoconiosis (16). Given the prolonged latency period characteristic of pneumoconiosis, our study specifically targets individuals aged ≥20 years—representing primary workforce contributors—to more accurately capture disease burden patterns in this occupationally vulnerable demographic.

Pneumoconiosis remains a substantial global burden, with persistent control gaps. Low-income and middle-income countries face constrained resources and weak occupational oversight, limiting enforcement. Rapidly industrializing nations often lack adequate worker protections during infrastructure development, coupled with weak health surveillance, incomplete regulations, and outdated laws. In high-income countries, lax monitoring of novel materials and legacy risks further adds to this burden. From 1990 to 2021, pneumoconiosis cases increased by ~20,705, accompanied by an annualized 0.48% decline in ASIR, indicating that global prevention measures have been largely effective in most regions.

Since 1990, multiple countries and regions worldwide have implemented strengthened policies and regulations to prevent the occurrence and progression of pneumoconiosis. The ILO/WHO *International Programme on the Elimination of Silicosis (IPES)*, launched in 1995, facilitated the development of national action plans in 30 countries (17). The European Union adopted the “Occupational Exposure Limit Directive” in 2004, establishing stringent controls for silica, asbestos, and other hazardous dusts (18). In addition, the inclusion of pneumoconiosis in workers' compensation insurance systems has been implemented across multiple jurisdictions, including Italy, France, Germany and so on (19). Collectively, these interventions have partially mitigated the pneumoconiosis burden and reduced ASIR.

Epidemiological analysis reveals that although Ukraine experienced the largest decline in the age-standardized incidence rate (ASIR) of pneumoconiosis, this trend must be carefully interpreted within its sociopolitical context. Since 2014, Ukraine has progressively adopted EU occupational safety standards by amending its *Labor Protection Law*, strengthening regulations in high-risk industries such as mining and construction (20), substantially decreasing the nation's ASIR of pneumoconiosis. However, actual implementation may be constrained by insufficient resources and the impact of conflicts. The armed conflict in eastern Ukraine in 2014 severely disrupted the public health system. Damage to medical facilities, shortages of healthcare workers, and the displacement of refugees may have undermined the capacity for pneumoconiosis prevention and treatment. Additionally, reduced industrial activity during the conflict might have temporarily decreased new cases of pneumoconiosis, but workers with long-term exposure still face the risk of disease progression. Most critically, the 2015 amendments to the Labor Code formally excluded temporary and seasonal workers from mandatory occupational health surveillance programs. Consequently, the reported decline in official pneumoconiosis cases may reflect systemic failures in Ukraine's diagnostic and monitoring infrastructure rather than a true reduction in disease prevalence (21).

Among the five SDI regions, only the high SDI region showed an ASIR increase from 1990 to 2021 (EAPC = 0.01), in contrast to the decline reported in GBD 2017 (22). The observed trend is likely to be driven by advancements in technologies in pneumoconiosis diagnostics, including the emergence of validated biomarker (e.g., KL-6, MMP-2) and the continuous evolution of AI-assisted imaging analysis systems for pneumoconiosis (23, 24). East Asia maintained the highest global pneumoconiosis ASIR in 2021, with persistently high incidence rates since 1990. China's silicosis-driven burden remains critical (2.72 per 100,000), thereby necessitating targeted policies to enforce occupational dust control in high-risk industries, particularly small enterprises. Australasia had the sharpest increase (EAPC = 2.72), driven predominantly by asbestosis in Australia, where disease latency spans 20–40 years. While WHO and the International Labor Organization (ILO) have issued global asbestos bans, persistent enforcement gaps necessitate strengthened national oversight to mitigate asbestos-related disease burdens.

Consistent with previous reports, males constitute 90% of miners and construction workers globally—occupations with prolonged exposure to silica, coal dust, and asbestos (25). This occupational segregation drives the disproportionately higher pneumoconiosis incidence observed in males compared to females. In 2021, cases peaked in the 65–69 age group, followed by 70–74 and 60–64 years, indicating delayed disease onset compared to 2019, suggesting that prevention and control measures have effectively delayed disease onset (25, 26). However, ASIRs were highest in those aged ≥85 years, aligning with accelerated aging populations globally.

Silicosis remains the most prevalent pneumoconiosis, accounting for 56.7% of cases in 2021, with over 70% occurring in East Asia and Southern Latin America. Silicosis, a progressive fibrotic lung disease caused by inhaling silica dust, has no cure and primarily affects workers in mining, construction, and manufacturing (27, 28). High-middle SDI region had the highest incidence, with China bearing the greatest burden, consistent with findings from the 2019 GBD study (27, 29). Occupational exposure remains significant due to hazardous work environments, inadequate protective measures, and emerging industries (30). Strengthened regulations, improved ventilation, and occupational health initiatives, such as China's *Healthy China 2030* plan, are essential for prevention (25, 31). What's more, the World Bank's (2017–2020) mining project reduced silica levels via German dust-control systems (32), paralleled by Belgium's EU-compliant silica/coal dust exposure limits enforced through penalties (33).

Asbestosis constituted 16.7% of cases, primarily in Australasia, High-income North America and Southern Sub-Saharan Africa. It was the only subtype with an increasing ASIR from 1990 to 2021. Asbestosis, a chronic interstitial pulmonary fibrosis caused by prolonged asbestos exposure, primarily affects workers in construction, shipbuilding, automotive repair and asbestos mining, with onset linked to the duration and intensity of exposure (34, 35). High SDI regions had the greatest burden, with Australia, High-income North America and Western Europe reporting high incidence rates due to prolonged asbestos exposure (34). While asbestos bans have reduced incidence in some countries, long asbestosis latency let the asbestos ban may not have an immediate effect. Additionally, stricter regulation, environmental controls and worker protection remain crucial (22).

CWP accounted for 11.9% of cases, with over 20% in the High-income Asia Pacific and Central Europe. Commonly referred to as “black lung disease,” CWP is caused by prolonged coal dust inhalation, primarily affecting coal miners and workers in related industries (36). In 2021, CWP burden was highest in high SDI and high-middle SDI regions, particularly in the High-income Asia Pacific and East Asia. The Republic of Korea and China faced the greatest burden, with workers exposed to coal dust in mining, transportation, and processing (37). Effective coal dust control, enhanced worker training, and protective measures have contributed to a decline in incidence. CWP accounted for 25.11% of global pneumoconiosis cases in 2017 (22), but incidence rates declined significantly by 2021, reflecting the effectiveness of safety measures. These trends highlight the importance of robust occupational health policies and preventive strategies in reducing CWP burden.

Other pneumoconiosis comprised 14.7% of cases, predominantly in Andean Latin America and Central Asia. This category includes conditions such as aluminum pneumoconiosis, berylliosis, siderosis and stannosis, in addition to silicosis, asbestosis, and CWP (22, 38). However, studies indicate that a majority of other pneumoconiosis subtypes are attributable to silicosis, asbestosis, or CWP, stemming from incomplete diagnostic coding and suboptimal categorization practices in source data (10). Misclassification may obscure industry-specific risks and misguide targeted prevention measures. From a statistical perspective, such systematic misclassification underestimates the true disease burden of these conditions, thereby diverting public health resources from critical needs, potentially leading to underprioritization in policy-making or miscalculated compensation schemes.

This study reveals a concerning upward trend in ASIR of pneumoconiosis across diverse national contexts—from traditional coal-producing nations like China to resource-rich countries like Australia where traditional mining coexists with emerging industries (e.g., engineered stone manufacturing). These concerning epidemiological patterns demand a multifaceted intervention strategy that addresses both primary and secondary prevention: at primary prevention, targeted interventions in high-incidence regions and vulnerable occupational groups by enforcing international standards (particularly ILO C155 Convention on Occupational Safety and Health), mandating engineering controls (wet processing, local exhaust ventilation) and protective supplies (N95 respirators masks), and adopting more stringent exposure limits for dust; enhanced secondary prevention measures including compulsory occupational health surveillance in high-risk sectors, incorporation of non-standard workers into monitoring programs, and strengthened regulatory oversight systems.

The observed ASIR elevation in high-SDI regions (2021 vs. 1990) likely reflects advancements in diagnostic capabilities rather than genuine epidemiological shifts, underscoring the importance of international collaboration to implement AI-assisted radiography interpretation for improved screening sensitivity while simultaneously building capacity among primary care providers to enhance early symptom recognition, particularly for chronic cough and pulmonary function decline, thereby minimizing diagnostic delays. Besides, the study reveals a significant correlation between robust legal frameworks and positive disease trends, as evidenced by nations with stringent judicial enforcement of occupational compensation systems (e.g., Italy, France, Germany) demonstrating substantial ASIR reductions, highlighting the critical need for implementing comprehensive legislated workers' compensation schemes specifically for pneumoconiosis coupled with rigorous compliance monitoring mechanisms to ensure effective policy implementation.

The successful implementation of this integrated approach–harmonizing technological innovation, policy transformation and international collaboration–promises to revolutionize resource allocation paradigms by simultaneously incorporating precision targeting of high-risk populations, equitable coverage of vulnerable worker groups, and sustainable prevention-oriented methodologies, collectively contributing to meaningful reduction of the global pneumoconiosis burden. This strategic framework not only addresses immediate diagnostic and therapeutic challenges but also establishes durable systemic solutions through institutionalized worker protections and continuous monitoring systems, thereby offering a viable pathway toward sustained mitigation of this preventable occupational health crisis across diverse industrial and developmental contexts.

This study has several limitations. First, the GBD 2021 dataset integrates data from diverse sources, inheriting inherent GBD biases such as selection bias and misclassification (22), potentially underestimating burdens of silicosis, asbestosis, and CWP while overestimating other subtypes, distorting etiological patterns and resource allocation. While we quantified 95% UIs and used DisMod-MR for missing data, future work requires standardized coding and weighting to reduce regional data-quality disparities. Second, GBD accuracy is constrained by data gaps, particularly in LMICs with weak occupational health surveillance (25, 26), resulting in underreported cases. Future iterations could integrate GBD's macro-level estimates with localized high-risk surveillance data to correct region-specific biases. Despite these limitations, GBD's comprehensive dataset provides valuable guidance for pneumoconiosis prevention and policy-making. We anticipate future GBD releases will incorporate more refined data sources to better inform disease-specific prevention strategies.

## 5 Conclusion

Pneumoconiosis remains a major global occupational health risk, with silicosis being the most common form. From 1990 to 2021, the global ASIR of pneumoconiosis among individuals aged ≥20 years declined, reflecting significant progress in preventive measures. However, high burdens persist in Australasia and East Asia, necessitating targeted action. Our findings provide policymakers with updated global burden data and region-specific evidence to enhance occupational health interventions. Future approaches must combine technology and regulation, prioritizing asbestos bans and lifelong monitoring in high-risk, male-dominated industries.

## Data Availability

The datasets presented in this study can be found in online repositories. The names of the repository/repositories and accession number(s) can be found in the article/supplementary material.
