# Supplementary material for: Global, regional, and national trends in the incidence of pneumoconiosis among populations aged 20 and above from 1990 to 2021
Source: Front Public Health. 2025 Aug 18;13:1608109. doi: 10.3389/fpubh.2025.1608109 (PMC12400962; doi:10.3389/fpubh.2025.1608109)
Supplement: Supplementary file 2 [file Data_Sheet_1.docx]

**Supplemental Information**

**The PDF file includes:**

Figure S1. Incident cases of pneumoconiosis attributed to various etiologies in different SDI regions from 1990 to 2021.

Figure S2. The correlation between EAPC and silicosis ASIR in 1990 (A), and HDI in 2021 (B). Figure S3. The correlation between EAPC and asbestosis ASIR in 1990 (A), and HDI in 2021 (B).

Figure S4. The correlation between EAPC and coal workers pneumoconiosis ASIR in 1990 (A), and HDI in 2021 (B).

Figure S5. The correlation between EAPC and other pneumoconiosis ASIR in 1990 (A), and HDI in 2021 (B).

Figure S6. The global disease burden of pneumoconiosis caused by silicosis in 2021 for individuals aged 20 and above.

Figure S7. The global disease burden of pneumoconiosis caused by coal workers pneumoconiosis in 2021 for individuals aged 20 and above.

Figure S8. The global disease burden of pneumoconiosis caused by asbestosis in 2021 for individuals aged 20 and above.

Figure S9. The global disease burden of pneumoconiosis caused by other pneumoconiosis in 2021 for individuals aged 20 and above.

Other Supplemental Materials for this manuscript includes the following:

Table S1. The incident cases and ASIR of pneumoconiosis in individuals age≥20 in 1990 and 2021.

Table S2. The incident cases and rates of pneumoconiosis in individuals aged ≥20 in 2021.

Table S3. The change of pneumoconiosis cases caused by four epitology between 1990 and 2021 for individuals aged 20 and above.

Table S4. Human development index in 2021, at national level.

Table S5. The incident cases and age-standardized incident rates of pneumoconiosis caused by silicosis in 1990 and 2021 for individuals aged 20 and above.

Table S6. The incident cases and age-standardized incident rates of pneumoconiosis caused by coal workers pneumoconiosis in 1990 and 2021 for individuals aged 20 and above.

Table S7. The incident cases and age-standardized incident rates of pneumoconiosis caused by asbestosis in 1990 and 2021 for individuals aged 20 and above.

Table S8. The incident cases and age-standardized incident rates of pneumoconiosis caused by other pneumoconiosis in 1990 and 2021 for individuals aged 20 and above.


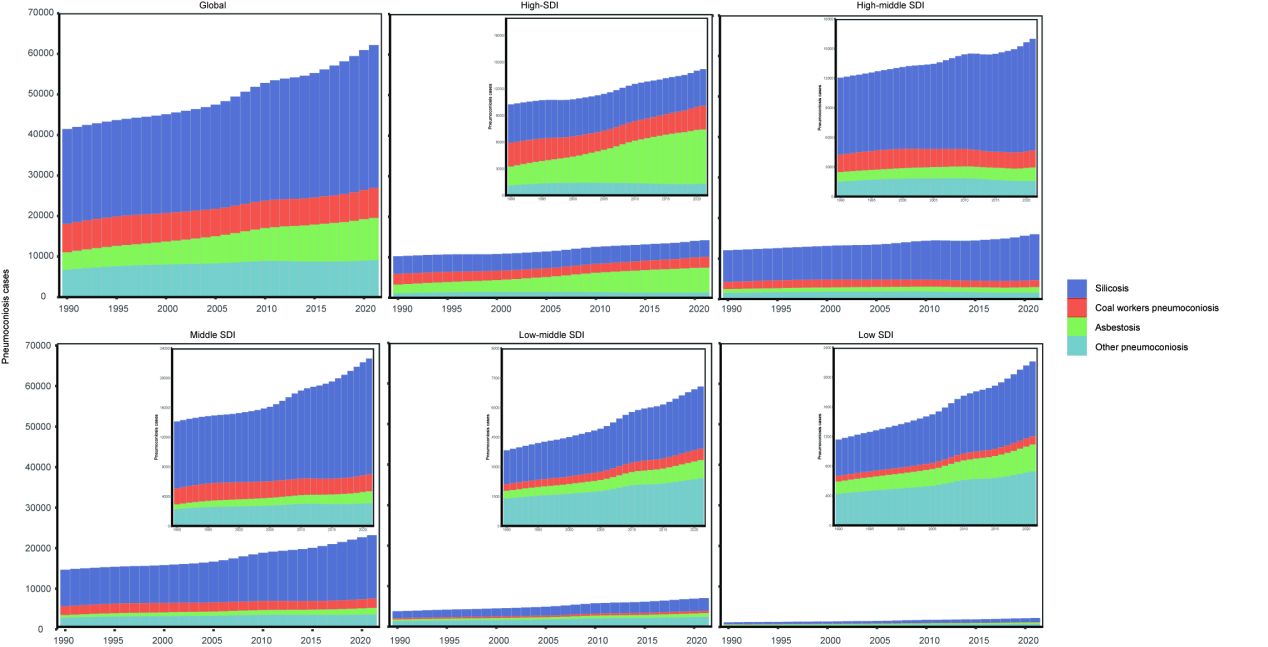


Figure S1. Incident cases of pneumoconiosis attributed to various etiologies in different SDI regions from 1990 to 2021.


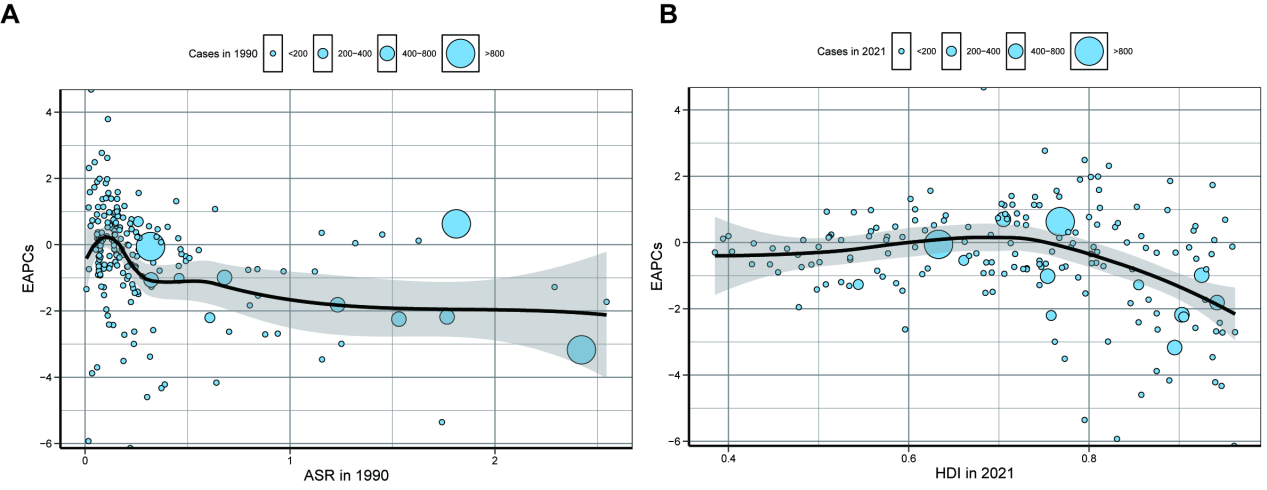


Figure S2. The correlation between EAPC and silicosis ASIR in 1990 (A), and HDI in 2021 (B). The circles represent countries that were available on HDI data. The size of circle is increased with the cases of silicosis. The Pearson’s product-moment correlation data were t = -4.75, df = 202, p-value = 3.80e-06, 95% CI: -0.44 to -0.19, r = -0.32 (A) and t = -3.38, df =178, p-value = 8.87e-04, 95% CI:-0.38 to -0.10, r = -0.25 (B).


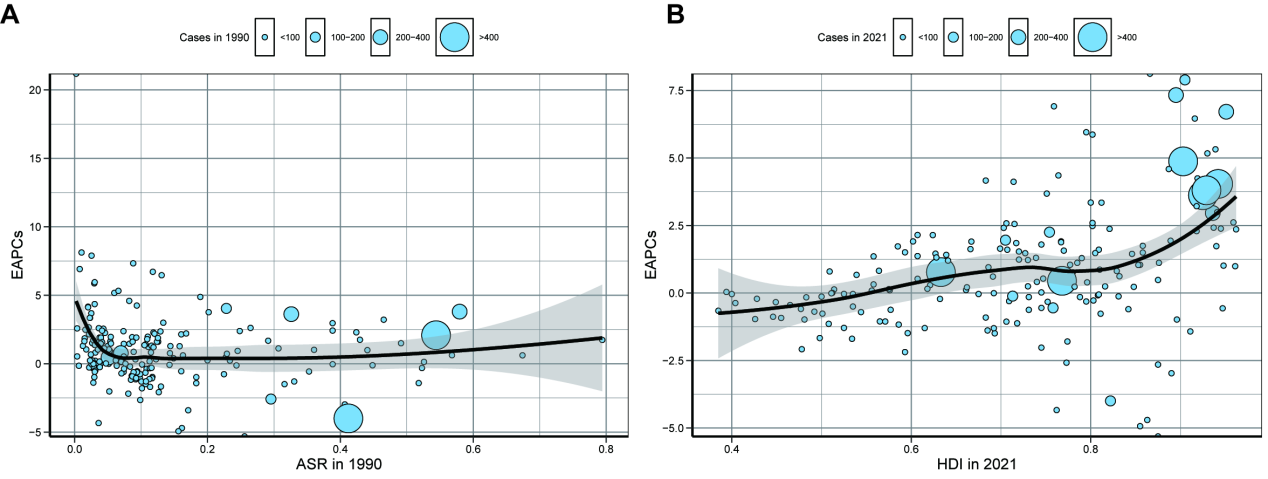


Figure S3. The correlation between EAPC and asbestosis ASIR in 1990 (A), and HDI in 2021 (B). The circles represent countries that were available on HDI data. The size of circle is increased with the cases of asbestosis. The Pearson’s product-moment correlation data were t = -1.45, df = 202, p-value = 0.15, 95% CI: -0.24 to 0.036, r = -0.10 (A) and t = 5.60, df = 178, p-value = 7.88e-08, 95% CI: 0.26 to 0.50, r = 0.39 (B).


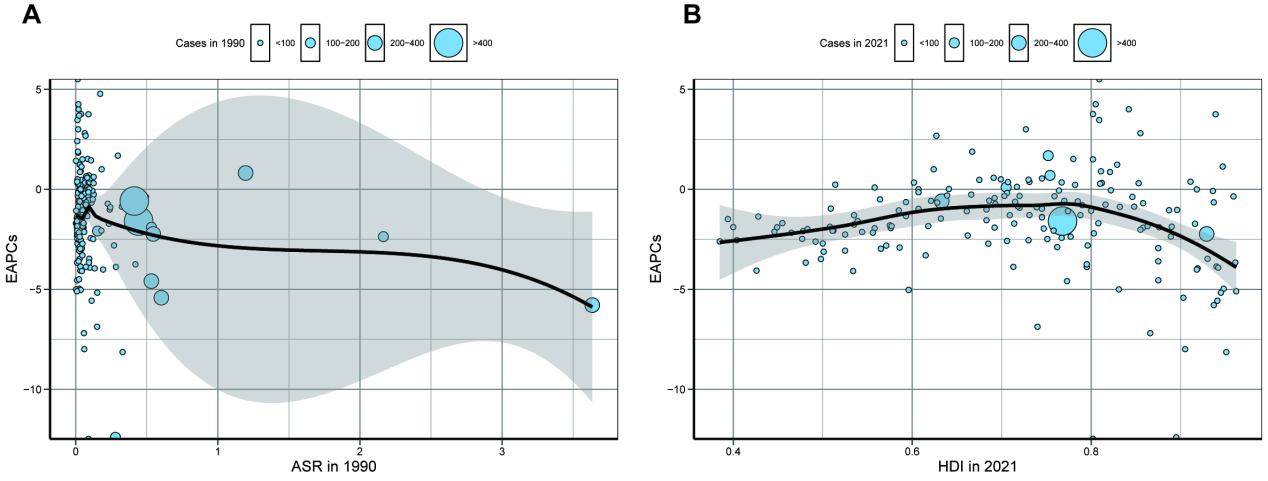


Figure S4. The correlation between EAPC and coal workers’pneumoconiosis ASIR in 1990 (A), and HDI in 2021 (B). The circles represent countries that were available on HDI data. The size of circle is increased with the cases of coal workers pneumoconiosis. The Pearson’s product-moment correlation data were t = -2.24, df = 202, p-value = 0.03, 95% CI: -0.29 to -0.02, r = -0.16 (A) and t = -0.77, df = 178, p-value = 0.45, 95% CI: -0.20 to 0.09, r = -0.06 (B).
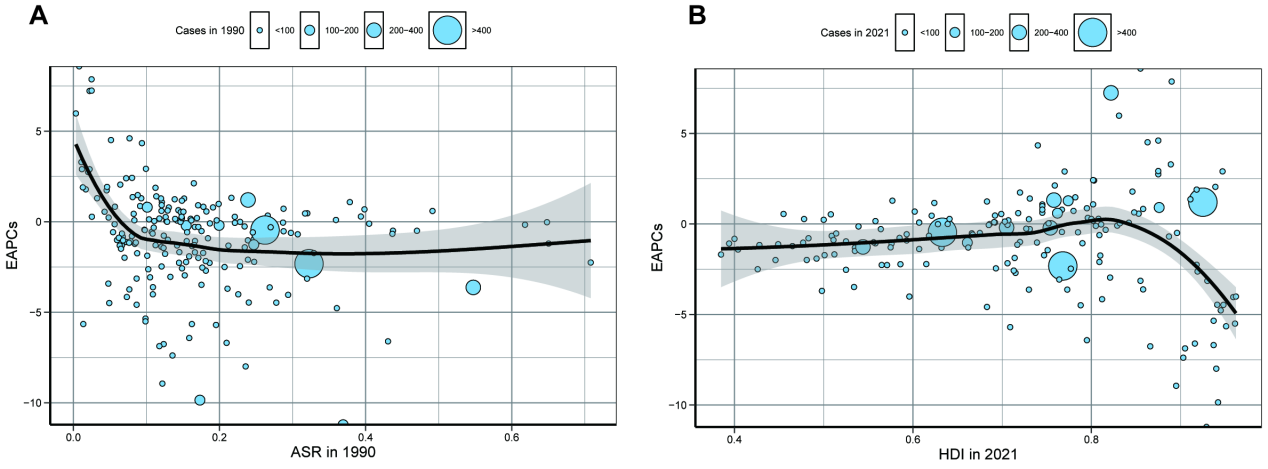


Figure S5. The correlation between EAPC and other pneumoconiosis ASIR in 1990 (A), and HDI in 2021 (B). The circles represent countries that were available on HDI data. The size of circle is increased with the cases of other pneumoconiosis. The Pearson’s product-moment correlation data were t = -3.37, df = 202, p-value = 9.11e-04, 95% CI: -0.36 to -0.10, r = -0.23 (A) and t = -1.00, df = 178, p-value = 0.32, 95% CI:-0.22 to 0.07, r = -0.07 (B).


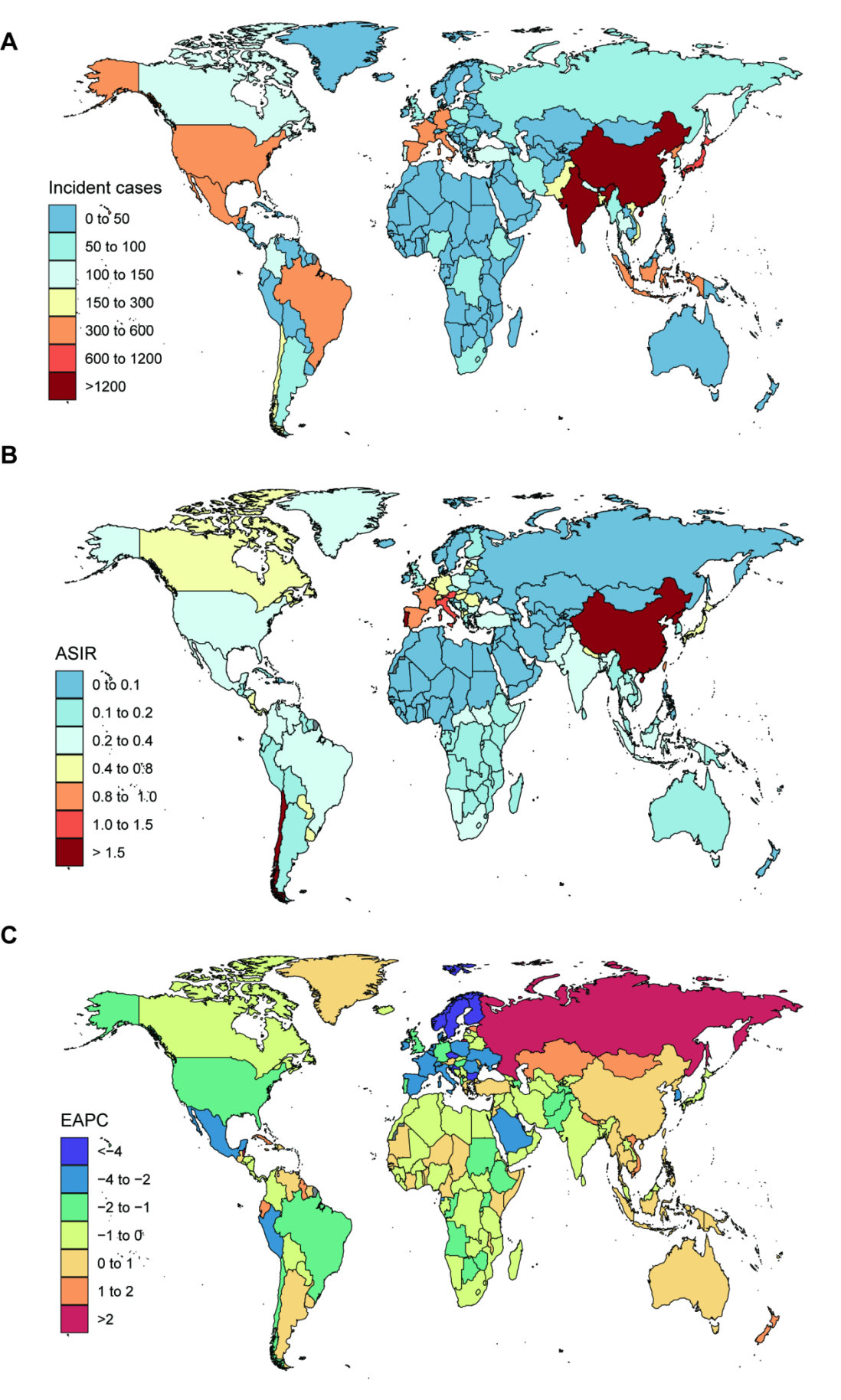


Figure S6. The global disease burden of pneumoconiosis caused by silicosis in 2021 for individuals aged 20 and above.

1. C) The incidence cases (A), the ASIR (B), and the EAPC (C) of pneumoconiosis caused by silicosis of individuals aged 20 and above in 2021. ASIR, aged standardized incidence rates; EAPC, estimated annual percentage change.


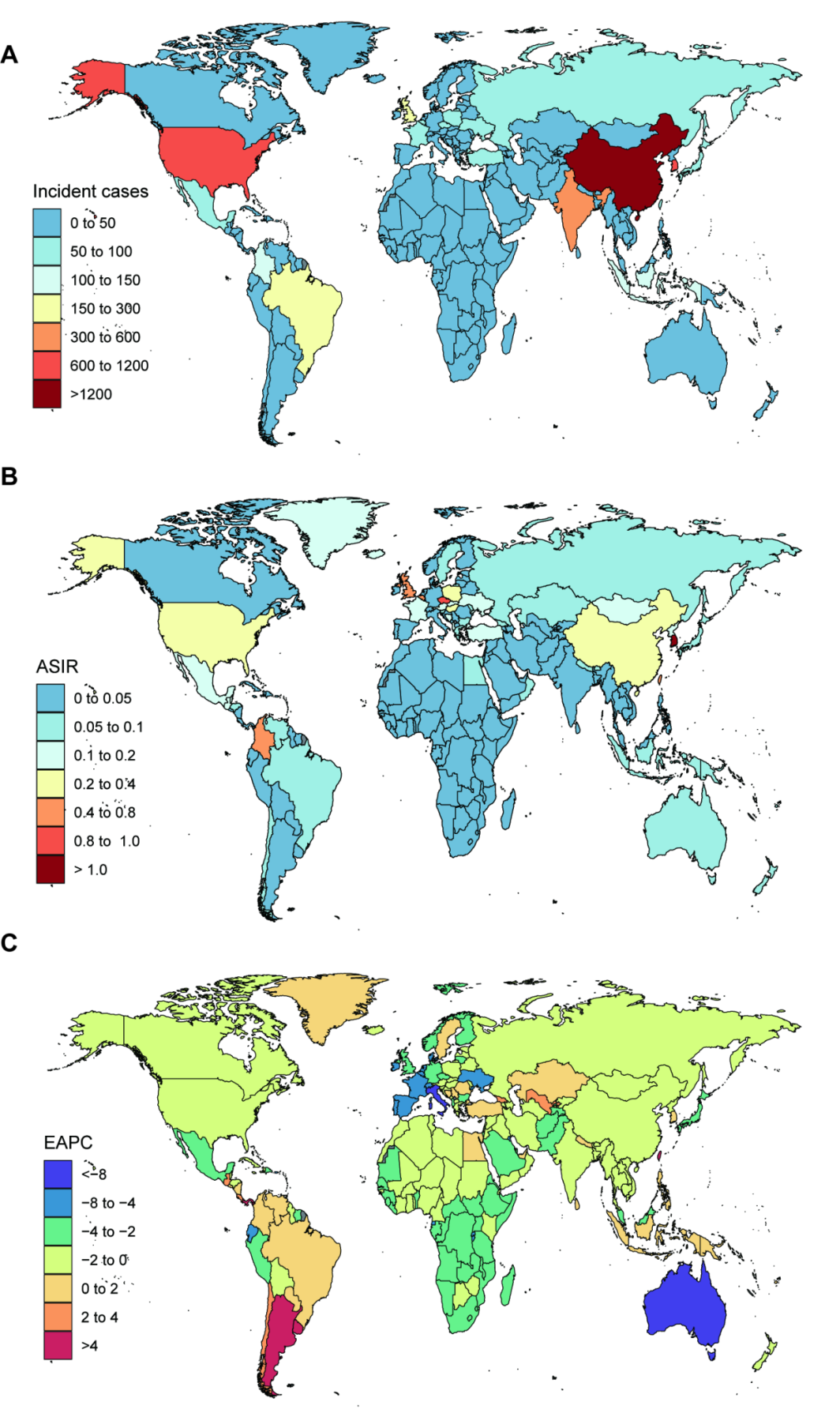


Figure S7. The global disease burden of pneumoconiosis caused by coal workers pneumoconiosis in 2021 for individuals aged 20 and above.

1. C) The incidence cases (A), the ASIR (B), and the EAPC (C) of pneumoconiosis caused by coal workers pneumoconiosis of individuals aged 20 and above in 2021. ASIR, aged standardized incidence rates; EAPC, estimated annual percentage change.


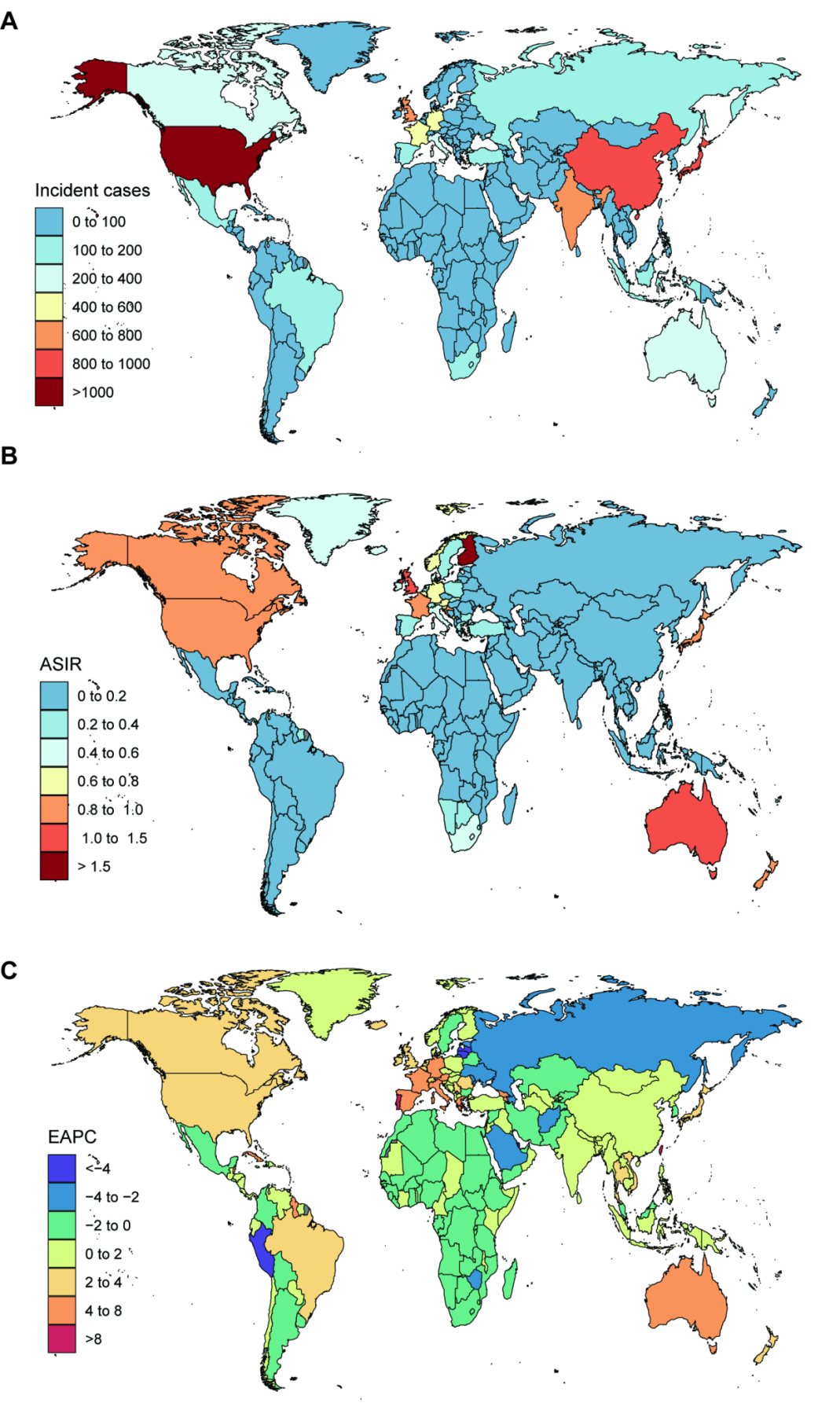


Figure S8. The global disease burden of pneumoconiosis caused by asbestosis in 2021 for individuals aged 20 and above.

1. C) The incidence cases (A), the ASIR (B), and the EAPC (C) of pneumoconiosis caused by asbestosis of individuals aged 20 and above in 2021. ASIR, aged standardized incidence rates; EAPC, estimated annual percentage change.


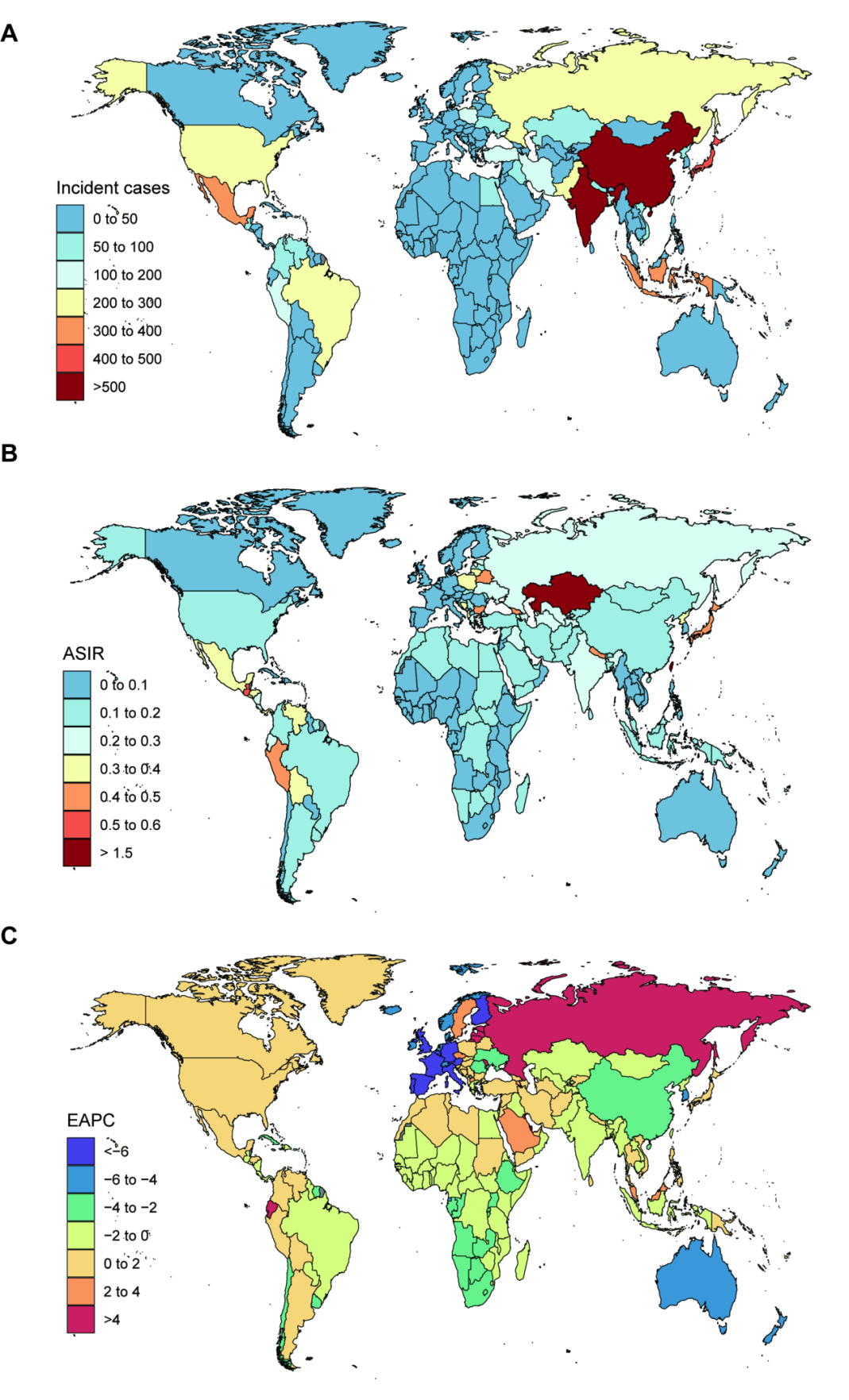


Figure S9. The global disease burden of pneumoconiosis caused by other pneumoconiosis in 2021 for individuals aged 20 and above.

1. C) The incidence cases (A), the ASIR (B), and the EAPC (C) of pneumoconiosis caused by other pneumoconiosis of individuals aged 20 and above in 2021. ASIR, aged standardized incidence rates; EAPC, estimated annual percentage change.
